# Synthesis and Characterization of ZnO/Chitosan Nanocomposites for Photocatalytic Degradation of Tetracycline in Water Media

**DOI:** 10.3390/polym18091114

**Published:** 2026-04-30

**Authors:** Phan Thi Minh Huyen, Luu Tien Hung, Phan Thi Hong Tuyet, Nguyen Huy Dan, Luu Thi Viet Ha, Tran Thi Nhu Quynh, Nguyen Xuan Dung

**Affiliations:** 1 College of Education, Vinh University, Vinh City 460000, Nghe An, Vietnam; huyenptm@vinhuni.edu.vn (P.T.M.H.); hunglt@vinhuni.edu.vn (L.T.H.); tuyetph@vinhuni.edu.vn (P.T.H.T.); tranthinhuquynh.study@gmail.com (T.T.N.Q.); 2 Institute of Materials Science, Vietnam Academy of Science and Technology, Hanoi 100000, Vietnam; dannh@ims.vast.ac.vn; 3 Faculty of Chemical Engineering, Industrial University of Ho Chi Minh City, Ho Chi Minh 700000, Vietnam; luuthivietha@iuh.edu.vn

**Keywords:** green synthesis, ZnO nanoparticles, ZnO/chitosan nanocomposites, photocatalytic degradation, tetracycline

## Abstract

Antibiotic contamination of water, particularly tetracycline (TC), poses significant environmental risks and requires sustainable treatment solutions. This study reports a green and cost-effective synthesis of a ZnO/chitosan nanocomposite (ZnO/CS) for photocatalytic TC removal. ZnO nanoparticles were synthesized using lime juice as a natural stabilizing agent and subsequently incorporated into a chitosan matrix. The physicochemical properties of the composite were characterized by X-ray diffraction (XRD), Fourier-transform infrared spectroscopy (FTIR), UV-Vis diffuse reflectance spectroscopy (UV-Vis DRS), scanning electron microscopy coupled with energy-dispersive X-ray spectroscopy (SEM-EDX), transmission electron microscopy (TEM), X-ray photoelectron spectroscopy (XPS), and Brunauer-Emmett-Teller (BET) surface area analysis. The results confirmed the successful formation of hexagonal wurtzite ZnO and strong interfacial interactions between ZnO nanoparticles and the –NH_2_/–OH functional groups of chitosan. The incorporation of chitosan significantly increased the specific surface area from 10.7 to 21.7 m^2^ g^−1^ and reduced the band gap from 3.18 to 3.03 eV, thereby improving visible-light absorption. The photocatalytic performance was evaluated under varying pH, initial TC concentration, and catalyst dosage, with optimal conditions identified at pH 6, 20 mg/L TC, and 1 g/L catalyst. Under these conditions, the ZnO/CS nanocomposite achieved 94.1% TC degradation within 120 min under visible-light irradiation. Scavenger experiments revealed that •OH and •O_2_^−^ radicals are the dominant reactive species, and a possible degradation mechanism was proposed. These findings demonstrate the potential of the green-synthesized ZnO/CS nanocomposite for antibiotic removal from aqueous environments.

## 1. Introduction

The increasing contamination of water resources by hazardous organic compounds, particularly pharmaceutical residues such as antibiotics, has emerged as a critical global environmental challenge owing to their adverse effects on aquatic ecosystems and potential risks to human health [1,2,3]. Tetracycline (TC) is an antibiotic extensively used in the pharmaceutical industry for humans as well as for livestock. When TC is released into the environment directly or indirectly, it causes various impacts on human health and the ecosystem [4,5,6].

Many techniques have already been implemented for water treatment such as membrane filtration, absorption, activated carbon, ozonation, ion exchange, Fenton catalytic reagent, electro-chemical destruction, etc. However, these techniques have some limitations, such as high cost, the formation of by-products, and the formation of strongly activated sludge, which is not suitable for wastewater treatment. Advanced oxidation processes (AOPs) by photocatalysts are considered a promising technique for wastewater treatment, which is based on the production of highly reactive oxidizing species that help to break down complex antibiotics bonds into simpler products [7,8]. Photocatalysts based on semiconductors have attracted great attention towards advanced material science research [9]. Among semiconductors, zinc oxide (ZnO) is widely used in various fields because of its distinct properties, such as a wide and direct band-gap energy, high exciton binding energy at room temperature, biocompatibility, abundant availability, good chemical stability, and low production costs [10]. These unique properties make ZnO a promising candidate for applications like ultraviolet light detectors, solar cells, transducers, sensors, and photocatalysts [10].

To synthesize ZnO nanoparticles (NPs), a variety of synthetic approaches have been carried out, such as hydrothermal processes, sol-methods, chemical vapor deposition, precipitations, laser ablations, and physical vapor depositions [11,12,13]. These methods typically involve the use of organic solvents and hazardous reducing agents, which are, in most cases, highly reactive and toxic to the environment [14]. Therefore, to overcome the challenges of this method, green synthesis could be an expectant alternative, enabling eco-friendly and cost-effective protocols using green and renewable materials [13]. Green approaches use biological resources such as plant extracts, bacteria, fungi, algae, and yeast, which contain natural phytochemicals that function as both reducing and capping agents for the synthesis of ZnO (NPs), providing an eco-friendly alternative to traditional chemical methods [14,15]. Lime, which is commonly used for cooking all over the world, has been employed for the synthesis of various nanomaterials owing to the high contents of bioactive compounds like ascorbic acid, citric acid, sugars, and polyphenols serving as reducing agents [16,17,18]. Therefore, the utilization of lime juice as a green synthesis medium represents a feasible and sustainable approach, as it not only replaces hazardous chemical reagents but also reduces energy consumption and environmental impact.

The fabrication of metal oxide-based composites has emerged as an effective route to improve the efficiency of semiconductor oxide photocatalytic systems. Hybrid composites formed by integrating polymers with metal oxides offer significant advantages by generating synergistic effects that enhance light absorption, reduce charge recombination, and improve photocatalytic performance [19,20]. Chitosan (CS), a natural polysaccharide polymer derived from chitin, has gained immense attention for its use in nanocomposite materials due to its biocompatibility, nontoxicity, biodegradability, and cost-effectiveness [21,22]. Additionally, chitosan’s numerous amino and hydroxyl groups make it an excellent adsorbent for heavy metal ions and other pollutant uptake [23].

The incorporation of oxide NPs into a chitosan matrix markedly enhances both the mechanical stability and photocatalytic activity of the resulting composite. The improvement in mechanical strength and structural integrity is mainly attributed to strong interfacial interactions between oxide and the functional groups (–NH_2_ and –OH) of chitosan, which reinforce the polymer network and effectively suppress nanoparticle agglomeration [24,25]. In parallel, the enhanced photocatalytic performance originates from the synergistic interaction between nanoparticles and the biopolymer matrix, where chitosan promotes uniform dispersion of active sites, facilitates interfacial charge transfer, and enhances pollutant adsorption via electrostatic and hydrogen-bonding interactions, leading to more efficient generation and utilization of reactive species under light irradiation [24,26].

Numerous studies have reported chitosan-metal oxide composites for environmental remediation, in which the metal oxide NPs are typically synthesized via established physicochemical routes before being incorporated into the chitosan matrix. For instance, ZnO NPs prepared by precipitation have been immobilized onto chitosan to form ZnO/CS nanocomposites for dye degradation and pesticide adsorption [27]. Similarly, TiO_2_ NPs synthesized through sol-gel method have been embedded in chitosan frameworks to develop photocatalytic membranes for water purification [28]. Furthermore, spinels (e.g., NiCo_2_O_4_, CoFe_2_O_4_) have been successfully loaded onto chitosan, yielding hybrid materials with enhanced adsorption capacity and photocatalytic performance toward various environmental pollutants [23,29]. However, most conventional strategies rely on chemical-intensive routes, potentially limiting the sustainability and scalability of these nanocomposites. Therefore, developing greener and more sustainable synthesis approaches that minimize chemical consumption and energy input is highly desirable for advancing chitosan-based functional materials for environmental applications.

In this study, green synthesis using lime juice was employed to prepare ZnO, followed by the fabrication of a ZnO/CS nanocomposite, in which ZnO NPs were uniformly immobilized on the chitosan matrix. The ZnO/chitosan composite was characterized and evaluated for its photocatalytic performance in tetracycline degradation.

## 2. Materials and Methods

### 2.1. Materials

All reagents were of analytical grade and used without further purification. Zinc acetate dihydrate (Zn(CH_3_COO)_2_·2H_2_O, 99%), chitosan (degree of deacetylation = 80%), and tetracycline (98%) were purchased from Sigma-Aldrich (St. Louis, MO, USA). Sodium hydroxide (NaOH, 98%), acetic acid (CH_3_COOH, 99%), and hydrochloric acid (HCl, 37%, d = 1.19 g/mL) were purchased from Aladdin Reagent Co., Ltd. (Shanghai, China). Deionized distilled water was used as the solvent throughout the experiment.

### 2.2. Collection of Lime Juice

Green lime was purchased from a supermarket in Vinh city, Nghe An province, Vietnam, and thoroughly washed with deionized distilled water. The juice was collected by squeezing the lime, followed by centrifugation (13,000 rpm, 10 min), filtration, and finally stored at 4 °C for further use.

### 2.3. Synthesis of ZnO/CS

A total of 1.097 g Zn(CH_3_COO)_2_·2H_2_O was dissolved in 50 mL of deionized water, followed by the addition of 20 mL lime extract and 30 mL deionized water. The mixture was magnetically stirred at 70–80 °C until gelation occurred. The resulting gel was dried and subsequently calcined at an appropriate temperature to obtain ZnO NPs.

For the preparation of ZnO/CS, 1 g of chitosan was first dissolved in 100 mL of 1% (*v*/*v*) acetic acid. Then, 0.3 g of the synthesized ZnO NPs was added to the solution. The mixture was magnetically stirred and ultrasonicated for 30 min. Then, 1 M NaOH solution was added dropwise until the pH reached approximately 10. The suspension was ultrasonicated for an additional 30 min, followed by heating at 80 °C for 3 h. Finally, the precipitate was filtered and washed several times with distilled water to reach a neutral pH, then dried in an oven at 50 to 60 °C for 6 h to obtain nanocomposite.

### 2.4. Characterizations of ZnO NPs and ZnO/CS Nanocomposite

The crystalline phases and structural parameters were determined by using X-ray Diffraction (XRD) on a D8 Advance diffractometer (Bruker, Karlsruhe, Germany) equipped with CuK_α_ radiation (λ = 0.15406 nm). The diffraction patterns were recorded in the 2*θ* range of 10° to 70°.

Surface functional groups and chemical interactions between ZnO and chitosan were identified using Fourier Transform Infrared Spectroscopy (FT-IR) on a Thermo-Nicolet Nexus 670 instrument (Thermo Fisher Scientific, Madison, WI, USA).

The surface morphology and particle size distribution were observed via Scanning Electron Microscopy (SEM, HITACHI S-4800, Hitachi, Tokyo, Japan) and Transmission Electron Microscopy (TEM, JEM-1400, JEOL Ltd., Tokyo, Japan).

Elemental composition and mapping were performed using Energy-Dispersive X-ray Spectroscopy (EDX, 7593-H, Horiba, Kyoto, Japan) integrated with the SEM system.

The optical properties and light-harvesting capacity of the solid samples were evaluated using UV-Vis Diffuse Reflectance Spectroscopy (UV-Vis DRS) on a Cary 5000 UV-Vis-NIR spectrometer (Agilent Technologies, Santa Clara, CA, USA).

The chemical valence states and surface elemental oxidation states were analyzed using X-ray Photoelectron Spectroscopy (XPS) on an ESCALab 250 spectrometer (Thermo VG Scientific, East Grinstead, UK).

The specific surface area and porosity of the materials were determined by the Brunauer-Emmett-Teller (BET) method using nitrogen adsorption-desorption isotherms on a Tristar-3000/Tristar-3020 analyzer (Micromeritics, Norcross, GA, USA).

### 2.5. Photocatalytic Evaluation

A 30 W LED lamp was used to provide visible light for all degradation experiments in this study. Photocatalytic assessments were performed in a glass vessel containing 50 mL of TC solution (50 mg/L) and 0.05 g of catalyst material. The TC solution containing the photocatalyst was magnetically stirred in the dark at room temperature for 60 min to achieve adsorption-desorption equilibrium. Then, the degradation performance of TC was assessed under visible light using ZnO, CS, and ZnO/CS. During the photocatalytic degradation of TC, a small aliquot of the solution was withdrawn every 30 min and analyzed by a UV-Vis spectrophotometer to determine the concentration of the remaining pollutant. The removal efficiency (RE) of TC was calculated using the following equation:

(1) $$RE\%=\frac{C_{o}-C_{t}}{C_{o}}\times 100\%$$

Here, $C_{t}$ (mg/L) and $C_{o}$ (mg/L) correspond to the concentration of TC at time t and the initial concentration, respectively.

The photodegradation kinetics of TC were evaluated based on a pseudo-first-order model, as expressed in the following equation:

(2) $$Ln\frac{C_{o}}{C_{t}}=kt$$

where *k* and *t* are respectively the kinetic rate constant and irradiation time. The *k* value indicates the photocatalytic activity, and it was calculated by the linear plot slope.

The factors affecting photocatalytic degradation, such as solution pH, initial TC concentration, and catalyst dosage were examined. The point of zero charge (pH_PZC_) of the material was determined using a typical pH-drift method [30].

Additionally, scavenger tests were performed to identify the dominant reactive species. Specifically, p-benzoquinone (BQ, for •O_2_^−^), isopropanol (IPA, for •OH), ethylenediaminetetraacetic acid (EDTA, for h^+^), and silver nitrate (AgNO_3_, for e^−^) were separately added to the reaction system. The experiments were conducted under identical conditions, with an initial TC concentration of 20 mg/L, pH = 6, and a catalyst dosage of 1 g/L at room temperature.

The recyclability and stability of the ZnO/CS nanocomposite were evaluated over four consecutive cycles. After each cycle, the catalyst was recovered by centrifugation, washed, and dried under vacuum at 80 °C for 5 h before being reused in a fresh TC solution for the subsequent run.

## 3. Results

### 3.1. Synthesis and Characteristics of ZnO NPs

**Figure 1:**
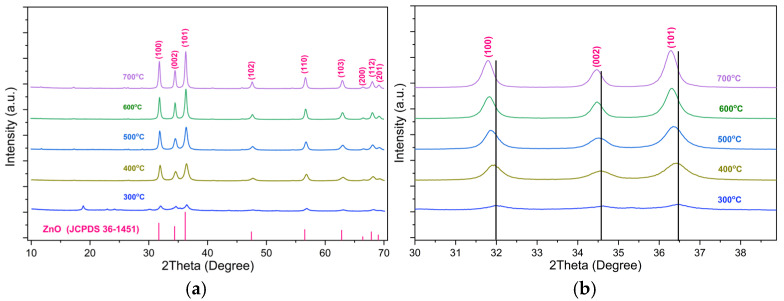
(**a**) XRD patterns of as-prepared and annealed ZnO NPs at 300 °C, 400 °C, 500 °C, 600 °C, and 700 °C. (**b**) Shifting of prominent peaks for (100), (002), and (101) crystal planes towards lower diffraction angle (2θ) with the increasing annealing temperature for as-prepared and annealed ZnO-NPs at 300 °C, 400 °C, 500 °C, 600 °C, and 700 °C.

To evaluate the crystallization process and the formation of the desired phase for ZnO, the sample was annealed at different temperatures and then analyzed by XRD (Figure 1). The sample heating in the temperature of 300 °C indicated broad and low peaks, proving that the sample is primarily in the amorphous form. With increasing calcination temperature (>300 °C), the samples gave the characteristic peaks of ZnO corresponded to the characteristic spacing between (100), (002), (101), (102), (110), (103), (200), (112), and (201) crystal planes of a hexagonal structure, providing clear evidence of the formation of ZnO (JCPDS number 36-1451) [31]. Furthermore, the reflection peaks became sharper with increasing annealing temperature, indicating an increase in crystallinity. From the above results, we chose the heating temperature of 500 °C to prepare ZnO NPs for the next experiments.

The average particle size D (nm) has been calculated using the well-known Scherrer’s formula [32]:

(3) $$D=\frac{K\lambda }{\beta \cos\theta }\;$$

where *K* is the correction factor, which is set as 0.9, and *β* is the Full Width at Half Maximum (FWHM) of the most intense peak (101).

In the ZnO hexagonal structure, the plane spacing d is related to the lattice constants *a*, *c*, and the Miller indices by the following relation [33]:


(4) $$\frac{1}{d_{hkl}^{2}}=\frac{4}{3a^{2}}\left(h^{2}+k^{2}+hk\right)+\frac{l^{2}}{c^{2}}$$

The lattice parameter (*a*, *c*), cell volume (V), and the X-ray density (*ρ*) were calculated from the (002) and (100) planes of XRD patterns using the following equations [33]:

(5) $$a=\sqrt{\frac{4d_{001}^{2}}{3}}\;$$

(6) $$c=2d_{002}$$

(7) $$V=\frac{\sqrt{3}}{2}a^{2}c$$

(8) $$\rho =\frac{nM}{NV}$$

where *M* is the molecular weight of the substance (81.38 g mol^−1^ for ZnO), *n* is the number of formula units in the unit cell (*n* = 2 for ZnO), and N is Avogadro number.

Table 1 lists the crystallite size (*D*), lattice parameter (*a*), cell volume (*V*), and the X-ray density (*ρ*) of ZnO nanoparticles at different annealing temperatures obtained from X-ray diffraction data.

It is evident that the calculated crystallite sizes are found to increase with the annealing temperature, which may be due to the merging of smaller crystallites into larger ones because of high annealing temperatures [31].

The calculated lattice parameters are in close agreement with those of bulk ZnO (JCPDS Card no. 36-1451): *a* = 3.2490 Å, *c* = 5.2060 Å. A progressive increase in annealing temperature leads to an expansion of the lattice constants ‘*a*’ and ‘*c*’ as well as the unit cell volume ‘*V*’, which can be attributed to the growth of crystallite size. Similar observations have been reported in previous studies [31,34,35].

In addition, the most prominent diffraction peaks of (100), (002), and (101) crystal planes are shifted towards lower diffraction angle (2θ) with increasing annealing temperature, which is evident from Figure 1b. This leads to an increase in interplanar distance of the ZnO crystal. This kind of behaviour can be attributed to a decrease in microstrain in the ZnO lattice after annealing [31,36].

### 3.2. X-Ray Diffraction Analysis of ZnO/CS

**Figure 2:**
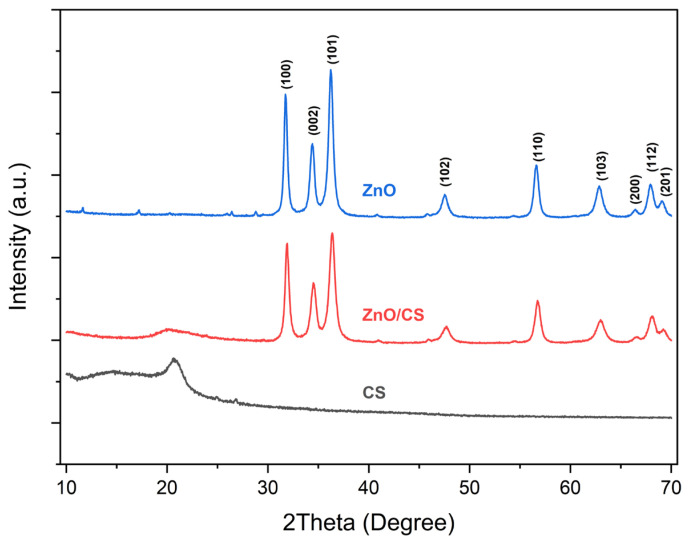
The XRD patterns of CS, ZnO/CS nanocomposite, and ZnO.

The XRD patterns of CS, ZnO, and ZnO/CS nanocomposite are shown in Figure 2. The XRD profile of CS clearly indicates a characteristic crystalline reflection at around 2*θ* ≈ 20°, which is typically associated with the semi-crystalline nature of chitosan [37,38,39].

For ZnO, the XRD pattern can be indexed to the wurtzite structure, which is well matched with the standard card for the hexagonal ZnO crystal (JCPDS card no. 36-1451). The diffraction peaks of ZnO were detected at 2*θ* values of 31.86, 34.53, 36.37, 47.67, 56.77, 62.97, 66.53, 68.07 and 69.23° corresponding to *hkl* values of (100), (002), (101), (102), (110), (103), (200), (112), and (201) [40].

In the case of the ZnO/CS system, the XRD pattern of ZnO/CS showed the main peaks corresponding to both ZnO and chitosan. However, the intensity of these peaks was lower than that of pure ZnO and chitosan, which can be attributed to interactions between ZnO and chitosan’s functional groups. It can be observed that the crystal structure of ZnO remained intact after interacting with chitosan, and the presence of two separate sets of diffraction peaks for ZnO and chitosan confirms the successful formation of ZnO/Chitosan [41].

### 3.3. FTIR Characterization

**Figure 3:**
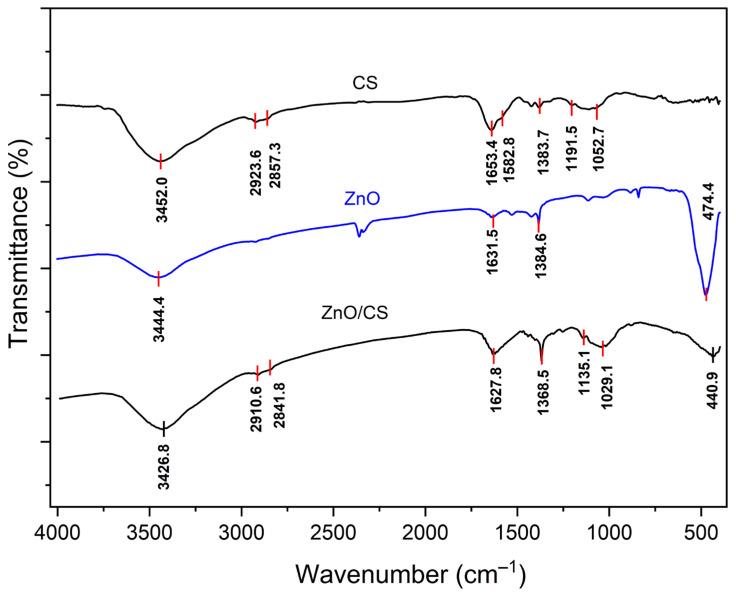
The FTIR spectra of pure CS, ZnO, and ZnO/CS nanocomposite.

Figure 3 shows the FT-IR spectra of pure CS, ZnO, and the ZnO/CS nanocomposite. In the CS spectrum, a broad peak at 3452.0 ${cm}^{-1}$ corresponds to the stretching vibrations of hydroxyl (–OH) and amino $({-NH}_{2}$) groups [42]. The absorption peaks at 2923.6, 2857.3 ${cm}^{-1}$ are attributed to the symmetric and asymmetric vibrations of C–H stretching vibration, respectively [43,44,45,46].

In addition, other absorption peaks appeared at 1635.4 ${cm}^{-1}$ corresponding to amide carbonyl groups, 1383.7 ${cm}^{-1}$ assigned to C–H bending vibrations, and 1052.7 ${cm}^{-1}$ attributed to the C–O–C stretching vibration of β-(1→4) glycosidic linkages connecting glucosamine and N-acetylglucosamine units in the chitosan polymer backbone [47,48,49,50]. The presence of free amino groups in deacetylated glucosamine mers is visible at 1582.8 and 1191.5 cm^−1^.

In the FTIR spectrum of ZnO NPs, the strong broad peaks in higher region at 3444.4 ${cm}^{-1}$ is due to the stretching vibration of hydroxyl (–OH) group on the surface of ZnO nanoparticle [51,52]. A slightly broad peak around 1633 ${cm}^{-1}$ is attributed to the bending vibration of the OH group, and might be due to the chemisorbed and/or physiosorbed moisture on the surface of ZnO NPs [53].

In the ZnO/CS nanocomposite, a combination of both CS and ZnO characteristic peaks was observed. The distinctive peaks of ZnO are shifted to a lower wavenumber, from 474.4 ${cm}^{-1}$ in pure ZnO to 440.9 ${cm}^{-1}$ in the ZnO/CS nanocomposite. Additionally, the broad peak at 3452.0 ${cm}^{-1}$, assigned to the stretching vibrations of the –NH_2_ and –OH groups in chitosan, shifted to 3426.8 ${cm}^{-1}$ in the ZnO/CS nanocomposite. The reason for this appears to be the interaction between ZnO molecules and chitosan’s OH and NH_2_ groups [54,55,56].

### 3.4. Microstructural and Elemental Analysis

**Figure 4:**
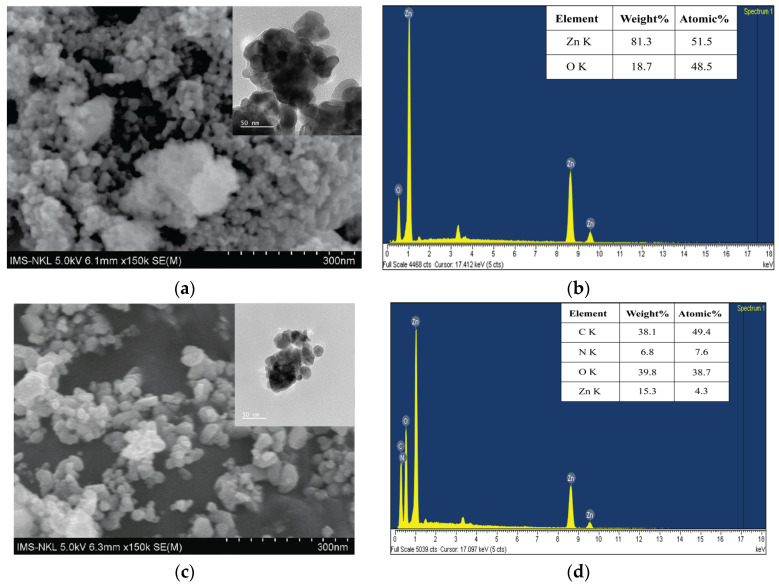
(**a**) FESEM and (**b**) EDS of ZnO NPs; (**c**) FESEM and (**d**) EDS of ZnO/CS nanocomposite (insets are the corresponding TEM images).

Figure 4 shows the FESEM, TEM images and the EDS profiles of ZnO NPs and ZnO/CS.

The SEM and TEM image (Figure 4a) shows ZnO nanoparticles with predominantly spherical to quasi-spherical morphology, with an average particle size of approximately 20–30 nm. The particles exhibit noticeable aggregation, forming larger agglomerates due to their high surface energy and interparticle interactions [57]. The SEM image of the ZnO/CS nanocomposite displays a significant change in morphology (Figure 4c). The original spherical shape of the ZnO nanoparticles is no longer clearly visible and appears to be embedded within or coated by the chitosan matrix. TEM image of ZnO/CS nanocomposite (Figure 4c) presented well defined and smoother particles. Thus, it can be concluded that chitosan prevented the known tendency of ZnO agglomeration. SEM and TEM images of ZnO/CS revealed that ZnO nanoparticles were uniformly distributed within the chitosan matrix with significantly reduced agglomeration compared to pure ZnO, suggesting that chitosan acts as a stabilizing and dispersing agent.

EDS analysis was performed to confirm the presence of different elements and approximate their composition in ZnO NPs and ZnO/CS nanocomposite (Figure 4b,d). EDS results indicated the presence of elements Zn, O in ZnO, and C, O, N, and Zn in the nanocomposite. The quantitative analyses, expressed as weight percentages for each element, showed good agreement with the expected values.

### 3.5. UV-VIS Diffuse Reflectance Spectroscopy (DRS) Analysis

**Figure 5:**
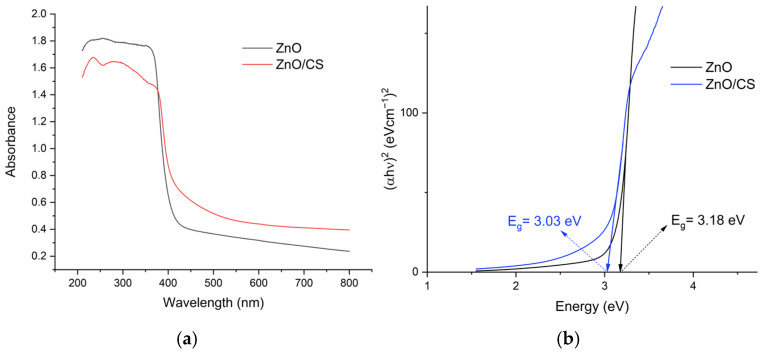
(**a**) UV-VIS diffuse reflectance spectrum of ZnO, ZnO/CS; (**b**) the corresponding Kubelka-Munk transformed reflectance spectrum.

Figure 5a shows the absorbance spectra of ZnO and ZnO/CS samples. There is a slight red shift in the UV-vis absorption edge, observed for ZnO/CS compared to ZnO. The optical band gap of the photocatalysts can be calculated from the plot of (αhυ)^2^ versus the photon energy (hυ) [58], as shown in Figure 5b. The band gaps of ZnO and ZnO/CS are 3.18 and 3.03 eV, respectively. The reduction in the optical bandgap of ZnO/chitosan nanocomposites compared to pure ZnO is primarily attributed to strong chemical interactions and complexation between Zn^2+^ ions and the amino (–NH_2_) and hydroxyl (–OH) groups of the chitosan matrix [59]. This interaction leads to the formation of interfacial heterojunctions and the introduction of intermediate energy levels within the forbidden gap, facilitating electronic transitions with lower energy requirements and enhancing light harvesting in the visible region [60].

### 3.6. X-Ray Photoelectron Spectroscopy (XPS) Analysis

**Figure 6:**
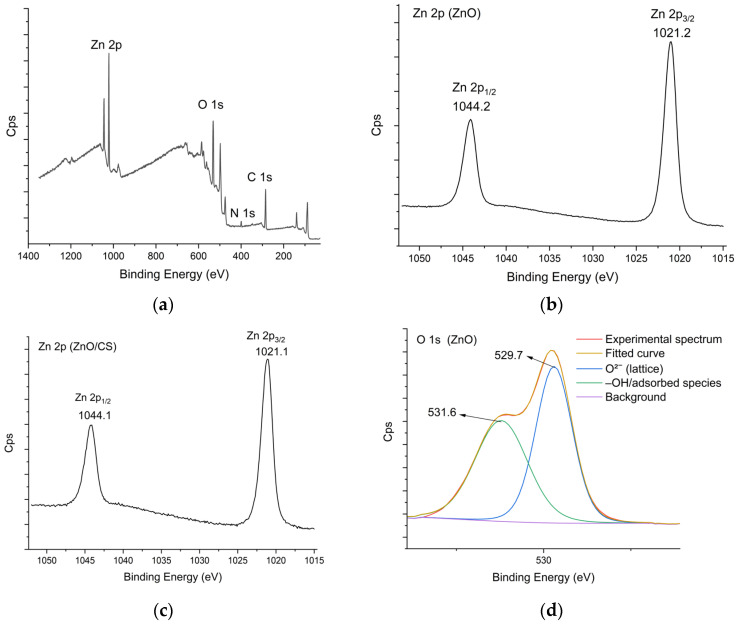

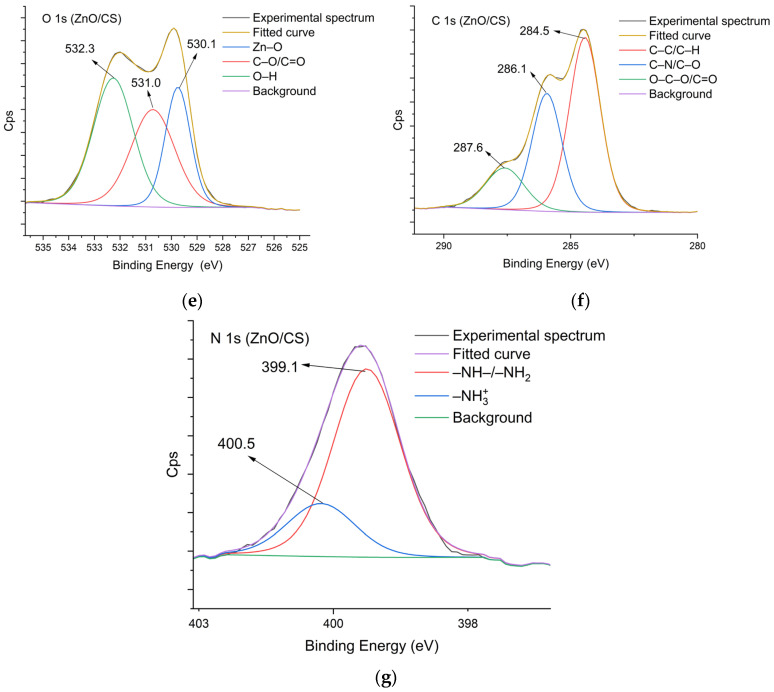
XPS spectra of ZnO/CS: (**a**) full range; (**b**) Zn 2p (ZnO); (**c**) Zn 2p (ZnO/CS); (**d**) O 1s (ZnO); (**e**) O 1s (ZnO/CS); (**f**) C 1s (ZnO/CS); (**g**) N 1s (ZnO/CS).

XPS was used to determine the electronic states of elements on the material surface. A full-range XPS curve of ZnO/CS is shown in Figure 6a, which confirms the presence of expected C, O, N, and Zn elements in the material.

For pure ZnO, the binding peaks at 1044.2 eV and 1021.2 eV, assigned to Zn 2p_1/2_ and Zn 2p_3/2_, respectively, confirm the presence of Zn^2+^ on the ZnO surface, as shown in Figure 6b. In the case of ZnO/CS, two corresponding peaks are observed at slightly lower energies of 1044.1 and 1021.1 eV (Figure 6c). This slight shift toward lower energy is attributed to the interaction between CS and ZnO nanoparticles [61].

The O 1s XPS spectrum of ZnO (Figure 6d) shows two peaks centered at 529.7 and 531.6 eV assigned to $O^{2-}$ ions in the ZnO lattice, and the water and/or the residual organic species coming from the precursors [62]. As displayed in Figure 6e, the O 1s spectrum of ZnO/CS was deconvoluted into three peaks and appeared at binding energies of 530.1 eV, 531.0 eV, and 532.3 eV, which were assigned to Zn–O, C–O/C=O, and O–H, respectively.

As shown in Figure 6f, the high-resolution XPS curve of carbon in the region C 1s exhibits four characteristic peaks at 284.5, 286.1, and 287.6 eV corresponding to the C–C/C–H, C–N/C–O, and O–C–O/C=O bond, respectively [63]. The N 1s high resolution spectra of ZnO/CS is shown in Figure 6g. The peaks at 399.1 and 400.5 eV were assigned to amino groups (–NH– and –NH_2_) and the protonated amino groups (–NH^3+^) from chitosan [64].

### 3.7. BET Analysis

The specific surface areas, total pore volume, and pore size of ZnO and ZnO/CS are provided in Table 2. As shown in Table 2, the incorporation of chitosan significantly influences the textural properties of ZnO. The BET surface area increases from 10.7 m^2^ g^−1^ for ZnO to 21.7 m^2^ g^−1^ for the ZnO/CS composite, indicating that chitosan effectively enhances the surface area. This enhancement can be attributed to the function of chitosan as a supporting matrix that promotes the homogeneous dispersion of ZnO nanoparticles and effectively suppresses their aggregation on the chitosan framework [61]. Similarly, the total pore volume rises from 0.012 to 0.11 cm^3^ g^−1^, while the average pore size decreases from 17.9 nm to 15.0 nm after chitosan incorporation, suggesting the development of a more porous structure upon composite formation. These results demonstrate that the ZnO/CS composite exhibits a more developed mesoporous structure, which is expected to provide a greater number of active sites and facilitate mass transfer during catalytic applications.

### 3.8. Photocatalytic Degradation

#### 3.8.1. Comparison of TC Removal Efficiency for ZnO and ZnO/CS

**Figure 7:**
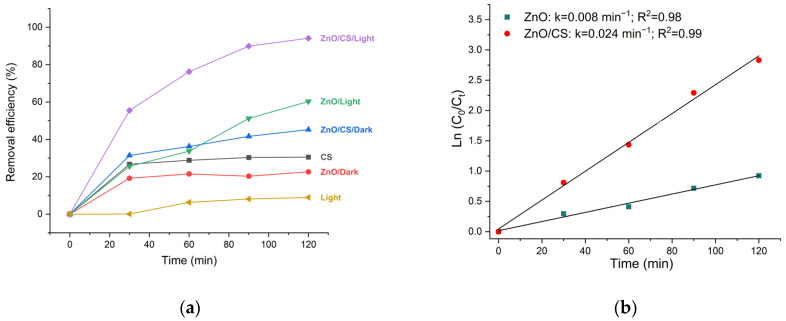
(**a**) Photocatalytic degradation of tetracycline (TC) over ZnO, and ZnO/CS. (**b**) Pseudo-first-order kinetic plots for ZnO and ZnO/CS.

The removal efficiency of TC by the samples is presented in Figure 7. Under dark conditions, the removal efficiencies of TC using CS, ZnO, and ZnO/CS are approximately 30%, 22%, and 45%, respectively. In the absence of the photocatalyst, the photodegradation of TC under light irradiation was very slow, reaching only about 10%.

After 120 min of light irradiation, the removal efficiencies of TC over ZnO and ZnO/CS were 60% and 94%, respectively. Moreover, the rate constant of ZnO incorporated chitosan is 0.024 min^−1^ in comparison to ZnO as 0.008 min^−1^. Therefore, the photocatalytic performance of ZnO has been enhanced with the addition of chitosan. The ZnO/chitosan nanocomposite achieves superior removal efficiency due to interactions between Zn^2+^ ions and the amino and hydroxyl groups of the chitosan matrix, which narrow the energy bandgap and extend light absorption into the visible range [50,59]. The incorporation of chitosan into the composite structure also effectively inhibits the recombination of photo-generated electron-hole ($e^{-}/h^{+}$) pairs, thereby extending the charge carrier lifespan to promote the generation of reactive oxygen species (ROS) such as hydroxyl radicals [50]. Additionally, chitosan acts as a substrate that promotes uniform nanoparticle dispersion, prevents agglomeration, and increases surface area, active adsorption sites, and photocatalytic degradation [50].

#### 3.8.2. Effect of Solution pH on TC Removal Efficiency

**Figure 8:**
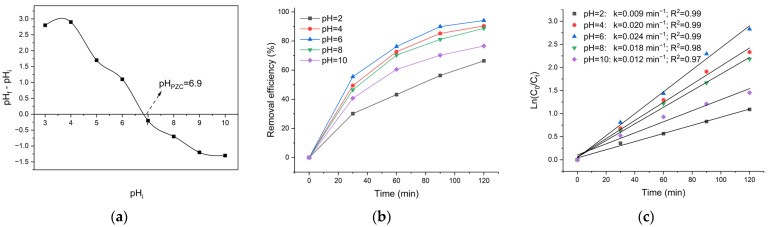
(**a**) Zero point charge (pH_PZC_) of ZnO/CS. (**b**) Effect of initial pH on the photocatalytic removal efficiency of TC. (**c**) Pseudo-first-order kinetic plots of TC degradation at different pH values.

The pH of the solution is a critical factor toward the photocatalytic efficiency [65]. The pH of the solution affects the surface charge of photocatalysts, the charge of pollutant species, and the surface adsorption of pollutant species. The pKa values of TC are 3.30, 7.68, and 9.70, indicating that TC exists in different charge states at different pH levels. Specifically, the predominant species are cation ($H_{4}{TC}^{+}$) at pH < 3.4, zwitterion ($H_{3}TC$) at 3.4 < pH < 7.6, anion (${H_{2}TC}^{-}$) at 7.6 < pH < 9.7, and other anion ($H{TC}^{2-}$) at pH > 9.7 [66,67]. The point of zero charge (pH_PZC_) is a property of solid particles in aqueous samples. pH_PZC_ provides essential insight into these interactions because it defines the pH at which the surface carries no net charge. Below the pH_PZC_, the surface becomes positively charged, while above it, the surface is negatively charged. As seen in Figure 8a, the pH_PZC_ of ZnO/CS nanocomposite is pH 6.9. At pH values below pH_PZC_, the ZnO/CS surface is positively charged, while it becomes negatively charged at pH values above pH_ZPC_.

Figure 8b clearly shows that the removal efficiency of TC increased from 66.4% to 94.1% with increasing pH from 2 to 6, then decreased to 88.7% and 76.6% with further increases in pH to 8 and 10, respectively. pH 6 is identified as the optimal pH for the photocatalytic degradation of TC by the material. In addition to removal efficiency, the rate constant reaches its maximum at pH 6, in agreement with the highest degradation efficiency observed under this condition (Figure 8c). The variation in degradation efficiency at different pH levels can be attributed to the electrostatic interactions between the surface of ZnO/CS and TC molecules. At pH = 2, both the ZnO/CS and TC materials are positively charged, leading to very poor interaction between the material and TC molecules. These make it difficult for the photocatalytic process to occur. At pH 4 and 6, TC molecules do not dissociate, and the ZnO/CS surface is positively charged, resulting in stronger interactions between the TC molecules and the material. Also, in pH > 7, ${H_{2}TC}^{-},{HTC}^{2-}$ species are dominant, and the nanocomposite surface is negatively charged. Thus, repulsion between the nanocomposite and the negatively charged TC species reduces the degradation efficiency. The findings of this study are consistent with previous reports on the photocatalytic degradation of TC [68].

#### 3.8.3. Effect of TC Concentration on TC Removal Efficiency

**Figure 9:**
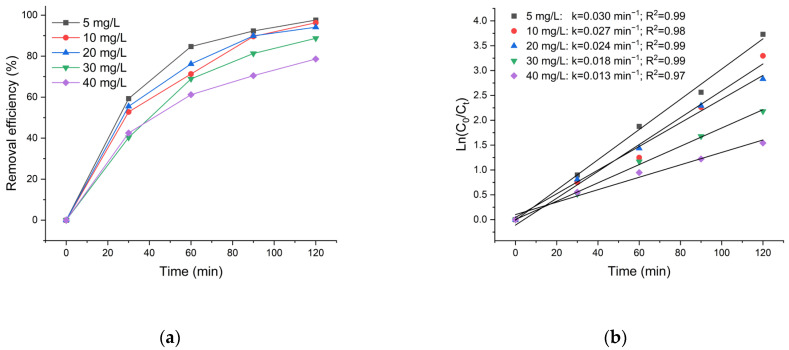
(**a**) Effect of initial TC concentration on the removal efficiency. (**b**) Pseudo-first-order kinetic plots of TC degradation at different initial concentrations.

The impact of tetracycline concentration on the photodegradation process was investigated by analyzing solutions with varying TC concentrations ranging from 5 to 40 mg/L (Figure 9a).

The results show that the highest removal efficiency (97.6%) was achieved at the lowest initial TC concentration of 5 mg/L. As the initial concentration increased to 10 and 20 mg/L, the photodegradation efficiency remained relatively high, reaching 96.3% and 94.1%, respectively. However, further increases in TC concentration to 30 and 40 mg/L resulted in a noticeable decline in efficiency, dropping to 88.7% and 78.6%, respectively. This trend can be attributed to the limited availability of active sites on the photocatalyst for degrading TC molecules at higher concentrations. As the TC concentration increases, the active sites on the catalyst surface become saturated, while excess TC molecules limit light penetration, leading to a decrease in photocatalytic degradation efficiency [69,70,71].

Figure 9b shows the kinetic study of the photodegradation of various concentrations of TC. The rate constants with initial concentrations of 5, 10, 20, 30, and 40 mg/L are 0.030, 0.027, 0.024, 0.018 and 0.013 min^−1^, respectively. The rate constant k decreased with increasing TCs concentration from 0.03 min^−1^ to 0.013 min^−1^. Therefore, the initial concentration of 20 mg/L was selected as the optimal condition. This value provides a balance between sufficient collision probability and minimized inhibitory effects, maintaining high degradation efficiency while avoiding surface saturation and excessive competition for active sites [72].

#### 3.8.4. Effect of Photocatalyst Dosage on TC Removal Efficiency

**Figure 10:**
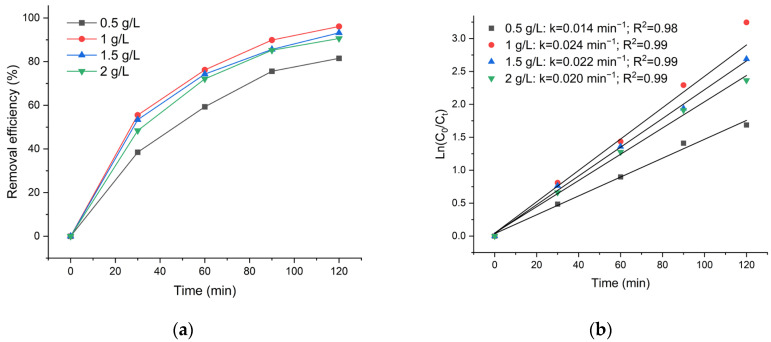
(**a**) Effect of catalyst dosage on the removal efficiency of TC. (**b**) Pseudo-first-order kinetic plots of TC degradation at different catalyst dosages.

Previous research confirms that an optimal catalyst dosage is essential for maximizing the photodegradation of pollutants like TC, and this value varies significantly depending on the specific nature and composition of the photocatalyst [68,73]. The impact of photocatalyst dosage on the photocatalytic degradation of TC was investigated using different amounts of nanocomposite catalyst ranging from 0.5 g/L to 2 g/L. The results are depicted in Figure 10.

The results show that increasing the ZnO/CS dosage from 0.5 to 1 g/L enhances the TC degradation efficiency from 81.5% to 94.1%, accompanied by an increase in the apparent rate constant from 0.014 to 0.024 min^−1^. This behavior is attributed to increased active sites and surface area, which promote adsorption, light harvesting, and ROS generation, leading to enhanced photocatalytic activity [68,74]. However, when the catalyst dosage exceeds 1 g/L, both the degradation efficiency and rate constant decline. Specifically, at dosages of 1.5 and 2 g/L, the degradation efficiency decreases to 93.2% and 90.6%, with corresponding rate constants of 0.022 and 0.020 min^−1^. This trend can be attributed to nanoparticle agglomeration and increased turbidity, which reduce active surface area and hinder light penetration, thereby limiting ROS generation [68,75]. Therefore, 1 g/L was identified as the optimal catalyst dosage in this study.

#### 3.8.5. The Recyclability Evaluation and Photocatalytic Mechanism

**Figure 11:**
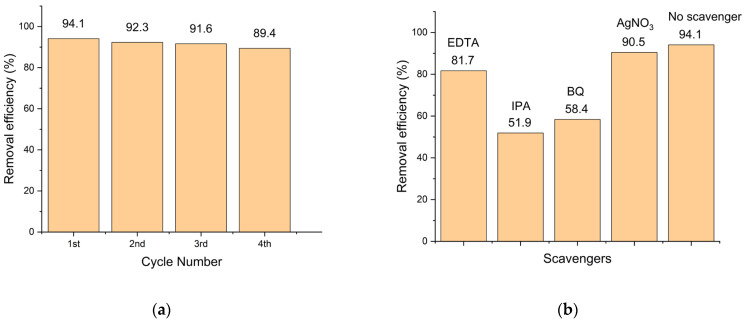
(**a**) Reusability of the ZnO/CS nanocomposite for photocatalytic degradation of TC. (**b**) The effect of scavengers (EDTA, IPA, BQ, and AgNO_3_) on the removal efficiency of TC.

Catalytic stability plays a crucial role in determining photocatalytic performance and contributes to cost reduction in the long-term treatment of organic contaminants. Figure 11a displays the TC elimination efficiency for each run. As shown in Figure 11a, the initial cycle achieved a removal efficiency of 94.1%, while the final cycle achieved 89.4%. The catalyst demonstrated remarkable photocatalytic activity and satisfactory reusability, as evidenced by the results. From the graph, the removal efficiency of antibiotics decreased by 4.7% in the fourth cycle compared to the initial cycle. This decline can be attributed to the presence of intermediate compounds, which compete with TC molecules for reaction with free radicals. Additionally, the pores and active sites of the catalyst may become blocked by both TC molecules and by-products. Extracting contaminants from the nanocomposite’s surface posed a challenge during recycling [66]. These experimental observations highlight the resilient crystal structure and chemically stable connection between ZnO and CS.

To gain a deeper understanding of the underlying photocatalytic mechanism, scavenger experiments were conducted to probe the roles of different reactive species. It is widely recognized that radical species are crucial determinants for photodegradation reactions. IPA, EDTA, BQ, and AgNO_3_ were utilized as selective scavengers for •OH, h^+^, •O_2_^−^, and e^−^, respectively. As illustrated in Figure 11b, the photocatalyst achieved a removal efficiency of 94.1% in the absence of scavengers. The addition of AgNO_3_ had a negligible effect on TC degradation, resulting in a reduction of less than 5%. This suggests that photogenerated electrons play a minimal role and are not the primary reactive species in the degradation process. The introduction of EDTA resulted in a greater decrease in degradation efficiency to 81.7%. Meanwhile, the presence of IPA and BQ led to significant reductions in efficiency to 51.9% and 58.4%, respectively. These results clearly demonstrate that •OH and •O_2_^−^ radicals are the dominant reactive species responsible for TC degradation over the ZnO/CS photocatalyst.

To elucidate the separation and migration of photogenerated charge carriers in ZnO/CS, the conduction band (CB) and valence band (VB) edge potentials were estimated using Mulliken electronegativity theory, based on the following empirical equations [60]:


(9) $$E_{VB}=\chi -E_{e}+{0.5E}_{g}$$

(10) $$E_{CB}=E_{VB}-E_{g}$$

In these formulas, χ represents the Mulliken absolute electronegativity of the semiconductor, which is 5.47 eV for ZnO [60]. The term $E_{e}$ stands for the energy of free electrons on the hydrogen scale, approximately 4.5 eV relative to the Normal Hydrogen Electrode (NHE). $E_{g}$ is the optical bandgap energy of the photocatalyst. Based on these calculations, $E_{CB}$ and $E_{VB}$ of ZnO/CS were determined to be −0.55 eV and +2.49 eV, respectively. These band potentials are crucial for evaluating the thermodynamic feasibility of ROS generation. Specifically, the CB potential (−0.55 V vs. NHE) is more negative than the standard redox potential of O_2_/^•^O_2_^−^ (−0.33 V vs. NHE), enabling the reduction of adsorbed O_2_ to superoxide radicals (^•^O_2_^−^). In addition, the sufficiently positive VB potential (+2.49 V vs. NHE) allows the oxidation of H_2_O or OH^−^ to hydroxyl radicals (•OH), as it exceeds the corresponding redox potentials (H_2_O/•OH: +2.38 V vs. NHE; OH^−^/•OH: +1.89 V vs. NHE) [66,68].

**Figure 12:**
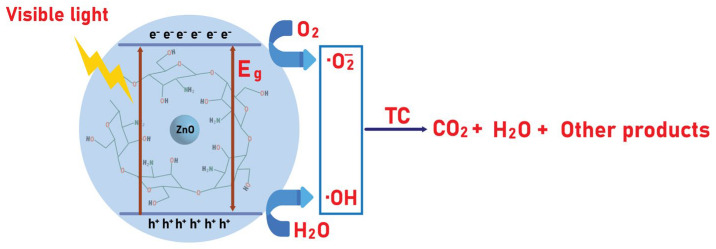
Mechanism of TC degradation via ZnO/CS.

Figure 12 illustrates the proposed mechanism of TC degradation over the ZnO/CS photocatalyst. Under visible light irradiation, ZnO/CS generates electrons (e^−^) and holes (h^+^) in the CB and VB, respectively. The photogenerated e^−^ reduce O_2_ to •O_2_^−^, while the h^+^ oxidize H_2_O/OH^−^ to •OH. The combined action of these species facilitates the degradation of TC into intermediates, which are subsequently mineralized into CO_2_, H_2_O, and other small molecules.

## 4. Conclusions

In this study, a green synthesis approach was successfully employed to fabricate ZnO nanoparticles using lime juice, followed by the preparation of a ZnO/CS nanocomposite with enhanced photocatalytic performance. Structural and spectroscopic analyses confirmed the successful integration of ZnO within the chitosan matrix and revealed strong interfacial interactions that improved the material properties. The ZnO/CS nanocomposite exhibited a higher surface area and a reduced band gap compared to pure ZnO, leading to enhanced visible-light absorption and photocatalytic activity.

The effects of key operational parameters, including solution pH, initial TC concentration, and catalyst dosage, were systematically investigated. The optimal conditions were identified as pH 6, an initial TC concentration of 20 mg/L, and a catalyst dosage of 1 g/L, under which the nanocomposite achieved a TC removal efficiency of 94.1% within 120 min under LED irradiation. The improved performance is mainly attributed to enhanced adsorption, better dispersion of ZnO nanoparticles, and more efficient separation of photogenerated charge carriers. Scavenger experiments revealed that •OH and •O_2_^−^ radicals are the dominant reactive species, and the material exhibited good stability and reusability over multiple cycles.

Overall, the ZnO/CS nanocomposite represents a sustainable, efficient, and low-cost photocatalyst with strong potential for practical applications in antibiotic-contaminated wastewater treatment.

## Figures and Tables

**Table 1:** The crystallite size (*D*), lattice parameter (*a*), cell volume (*V*), and the X-ray density (ρ) of ZnO nanoparticles at different annealing temperatures obtained from X-ray diffraction data.

Annealing Temperature (°C)	*D* (nm)	Lattice Parameters (Ả) *a* *c*		*V* (Ả^3^)	$\rho \;(\frac{g}{{cm}^{3}})$
300	6.5	-	-		-
400	11.2	3.2339	5.1852	46.96	5.75
500	13.9	3.2408	5.1910	47.22	5.72
600	18.7	3.2428	5.1983	47.34	5.71
700	21.6	3.2468	5.2012	47.48	5.69

**Table 2:** The BET surface area, pore volumes, and pore sizes of ZnO, and ZnO/CS.

Material	BET Surface Area (m^2^ g^−1^)	Pore Volume (cm^3^ g^−1^)	Pore Size (nm)
ZnO	10.7	0.012	17.9
ZnO/CS	21.7	0.11	15.0

## Data Availability

The original contributions presented in the study are included in the article. Further inquiries can be directed to the corresponding author.
